# Obscurity on Obesity

**DOI:** 10.1186/1741-7015-12-114

**Published:** 2014-08-26

**Authors:** Jack Winkler

**Affiliations:** London Metropolitan University, London, UK

**Keywords:** Obesity, Obesity policy, Weight gain, Weight loss, Diet surveys, Food intake, Nutrition education, Food taxes, Food regulation, Nutritional reformulation

## Abstract

Much research is underway on the links between diet and obesity. So too are long-running disputes among nutritionists on core questions about the relationship. This editorial reviews the state-of-play on four issues: what makes people fat, how to lose weight, how much do we eat, and what policies to adopt towards obesity. The practical consequence is that, at present, frontline health professionals will not find in nutrition science agreed, actionable solutions to assist overweight patients. But research and debate continues actively.

## Editorial

More than half of humanity suffers some serious problem with diet. In the Food & Agriculture Organization’s latest count [1], some 850 million are hungry. At the other end of the scale, 1.4 billion are overweight or obese. Another 2 billion-plus suffer micronutrient deficiencies.

Hunger has been part of the human condition from the beginning. So, too, in consequence, have shortages in some essential nutrients. But obesity, on a mass public health scale, is new.

Nutrition science is also relatively new, effectively a 20^th^ century creation. On the basics of what the human animal ought to eat, as expressed in dietary recommendations around the world, the experts are more or less agreed [2].

But on many specific issues there are long-running differences of view, often intense, even polemical, in character. Obesity is one area of conspicuous contention.

In part, these disputes reflect the youth of the science, in part the practical difficulties of conducting controlled research, lasting many years, on the daily routines of large numbers of free-living subjects.

The practical consequence is that the many non-nutritionists working on obesity have difficulty finding agreed, actionable conclusions from nutrition science on what to do about the problem.

Hence, it is appropriate that *BMC Medicine* should devote a special collection of articles to the latest obesity research. But it is also appropriate to set new findings in the context of on-going controversies. That is the purpose of this introduction.

It considers four disputed questions relevant to obesity, intentionally phrased in pragmatic terms. (1) What makes us fat? (2) How do we lose weight? (3) How much do we eat? (4) What policies should we adopt on obesity?

**What Makes Us Fat?** The orthodox position, for years, has been that people gain weight when they consume more calories than they expend in physical activity. Calories in exceed calories out.

But, increasingly, many argue that all calories are not created equal. Some nutrients lead to greater weight gain than others. Calorie counts are not enough. The source of those calories counts too. Quality as well as quantity.

Candidate Number One was fat. This is prima facie plausible. Fat provides nine calories per gram, protein and carbohydrates only four. Such reasoning stimulated a proliferation of low/reduced fat products, plus diets that emphasised eliminating fatty foods.

But even from the beginning there were dissenters, most famously John Yudkin [3], now with hindsight seen as a John the Baptist figure for Robert Atkins [4], who made carbohydrates a major focus in weight debates, and who in turn attracted many apostles.

Within carbohydrates, many have emphasised sugars rather than starches, because they are rapidly metabolised (have a ‘high glycemic index’), provoking a strong insulin response, leading eventually in some people to insulin resistance.

Sugars have recently become the principal focus of concern, through the powerful advocacy of Robert Lustig [5], the new lower consumption targets suggested by the World Health Organization [6] and in the UK by the publication of the draft report on carbohydrates by the Scientific Advisory Committee on Nutrition [7], and by the formation of a prominent new advocacy group, Action on Sugar.

And within the sugars, Lustig and others point particularly at fructose, which is metabolised differently than the rest.

These food fights have been supplemented by the revival of a parallel debate on an eternal problem: is it gluttony or is it sloth? Or both? Some blame the obesity epidemic in developed countries on increasingly sedentary lifestyles -- less manual work, numerous labour-saving appliances, television watching as the principal leisure activity, and whole lives spent, from an early age, seated in front of computers [8].

These disputes have been continuing for half a century and are intensifying rather than diminishing. If you want to know what makes us fat, there is a cornucopia of options to choose from [9].

And, all this is about to become much more complicated as we gradually discover more about the genetics of obesity. Are our genes out of sync with our new ‘obesogenic’ environment, dense with fast food takeaways and convenience stores (the Paleo Diet)?

Or, poignantly, are some individuals genetically disadvantaged, predisposed to weight gain? If so, that knowledge, too, is likely to breed more new diets (eat for your genes), sold on a pitch that is bound to prove popular (it’s not your fault!).

**How Do We Lose Weight?** In the current context, where the majority of adults in most developed countries are overweight/obese, the question of how to shed excess kilos/pounds is a matter of widespread concern. Many are keen to offer solutions, some serious, some sensational.

The world of weight loss is littered with faddish new regimes, ‘miracle diets’, ‘superfoods’ and ‘fat zappers’, stimulated by the prospect of sales as much as science. Popular media amplify the competing claims, regularly featuring testimonials from weight loss champions, or ‘Dieter of the Year' competitions.

Nowadays, promotions even promise that dieters need never go hungry, or that they can eat whatever they like --- at least for five days, so long as they fast for the other two.

Part of the problem is that ‘authors only cite the work that supports their point of view, ignoring a vast literature that refutes it’. As a result, ‘arguments about the best variant on that theme are mostly unfounded, sometimes utter nonsense…’ [10, 11].

One long-running example, currently topical again, concerns the benefits or otherwise of substituting sugar with sweeteners. For 20 years, two parallel but contradictory streams of research (so-called ‘compensation studies’) have flowed unabated. One concludes that sweeteners aid weight loss, the other that, perversely, they lead to weight gain. Faced with such ‘findings’, how are frontline practitioners to advise patients about diet/light/zero/max colas?

In theory, the effectiveness of weight loss regimes ought to be susceptible to scientific investigation, controlled experiments to see which works best. Indeed, there have been many ‘diet trials’.

There are many technical problems with such research, but the most fundamental is that it is impossible to measure whether participants actually followed the prescribed diets, to what degree, for how long, if at all [12].

Thus, comparisons between competing diets are imperfect at best. Arguments, pro and con, are based on biological first principles rather than empirical evidence. Plausible perhaps, but not proven.

**How Much Do We Eat?** The difficulties of measuring compliance with diets are one manifestation of a more fundamental problem in nutrition science – the inability to measure accurately what people actually eat.

Established methods involve two stages. First, there is an attempt to determine what foods people consume and how much. Then, the calorie and nutrient content of those foods is extracted by reference to a ‘compositional database’, which lists the average nutrient profiles of commonly consumed foods.

Several methods are used in ‘diet surveys’ – 24-hour recalls, recalls over several days, weighed food studies, food diaries, food histories, food frequency questionnaires.

All effectively rely on subjects telling researchers honestly what they eat. But they do not. These days, most people claim to eat a healthier diet than they actually do, smaller in volume and a more nutritious mix. In the trade, this is called ‘under-reporting’. In plain English, people lie.

These are not malicious lies. They are the ordinary lies we all tell every day – showing ourselves in the best possible light. So piffling are these fibs that many are happy to acknowledge them when asked [13]. Better to dissemble than admit eating ‘bad’ foods.

But inaccurate information on intakes undermines our ability to understand obesity and to take action against it. We cannot, for example, rigorously sort out the alternative theories about the basic question of what makes us fat.

Readers unfamiliar with diet surveys may at this point be asking themselves: can the science really be as bad as that?

Sadly, yes. The lies people tell about their food may be white lies, but they are large lies. In the UK, for example, which does better diet surveys than most, separate research using a biomarker, ‘doubly-labelled water’, showed that adults under-report their calorie intakes by 25% [14], late adolescents by 34% [15].

In one study of soft drinks, subjects in the National Diet and Nutrition Survey claimed to be drinking barely a quarter of the products that manufacturers reported they were selling [16].

In the US, a recent review of 39 years of the American national diet survey (National Health and Nutrition Examination Survey (NHANES)) found that ‘data on the majority of respondents (67.3% of women and 58.7% of men) were not physiologically plausible’ [17]. Subjects were claiming to eat less than is necessary to stay alive.

As a result, 17 leading obesity experts recently concluded, ‘Going forward we should accept that self reported energy intake is fatally flawed and we should stop publishing inaccurate and misleading energy intake data’ [18].

That is a rigorous assessment of the available science. But it does not help much ‘going forward’, that is, in deciding what we should do about obesity.

**What Obesity Policies?** In contrast to disputes about science and diets, there has been a remarkable level of agreement on public health policy to deal with nutritional problems, including obesity.

Three recent international surveys of nutrition policies [19–21] have found that, overwhelmingly, everywhere, the most common approach has been educational programmes directed at consumers. They exhort people to choose different foods and provide them with basic facts on which to make ‘informed healthy choices’.

Most nutrition policies share another characteristic -– they have failed. In most countries, developing and developed alike, with men and women, adults and children, rich and poor, people grow fatter and fatter. We must be doing something wrong.

The positive side of this failure is that in recent years, public health specialists have been willing to consider a wider range of options. The dish-of-the-day in obesity policy these days are proposals to tax ‘bad’ foods, especially soft drinks.

The practical problems are that such remedies are unlikely to be adopted and would not make much difference if they were.

The most serious study ever done on UK food purchasing showed that a 10% tax on soft drinks would reduce consumption by 7.5 ml per person per day – less than a sip [22].

In addition, the politics of policy intrudes. In the UK, only two years ago, there was a popular revolt against a small ‘pasty tax’. The government backed down. In Denmark, one of the most tax-tolerant countries on Earth, the government introduced a ‘fat tax’, then repealed it after a year because of near universal opposition. After those experiences, no democratically elected politician is likely to touch food taxes for years.

Many see increased obesity as caused by increased consumption of processed foods. In developed countries like Britain, some 70% to 85% of energy intake comes from manufactured products. Their nutritional quality shapes the nutritional status of nations.

Understandably, therefore, many propose regulating the composition of foods. But the political climate has changed. These days, most western governments no longer see regulation as a form of consumer protection, but as a ‘burden’ on business. They favour ‘light touch’ regulation, fewer rules not more.

Education, taxation, regulation. These have long been the principal instruments for public health in many fields. With food, for the foreseeable future, all are economically ineffective, politically unacceptable, or both.

More recently, attention has turned to the possibility of reformulating those popular processed foods. Partly, this has been inspired by two successful precedents.

In the UK, the programme to reformulate the 80+ most important salt-bearing products has reduced average national salt consumption by 15% in just six years [23]. In the US, 16 leading food manufacturers have cut 6.4 trillion calories from their sales in three years [24].

A theoretical logic underlies this approach. If people will not choose different foods, start from the foods that they actually eat, then improve their nutrient profiles. If we cannot change people, then change foods instead.

This has been an overview of some of the current issues within nutrition science. Research at present does not provide ready-made solutions to plug in for obesity problems. But science always looks tidier in retrospect than during a discovery phase.

The optimistic reading is that obesity research is an extremely active field, with many engaged, passionately. And so they should be. Obesity is an extremely important problem. We certainly need more and better research to cope with it. This collection of articles in *BMC Medicine* is a contribution to understanding the major public health problem of our age.

## Author’s information

JTW was formerly Professor of Nutrition Policy at London Metropolitan University.
